# Implant angulation and fracture resistance of one-piece screw-retained hybrid monolithic zirconia ceramic restorations

**DOI:** 10.1371/journal.pone.0280816

**Published:** 2023-01-23

**Authors:** Milad Bonyatpour, Rashin Giti, Behrad Erfanian

**Affiliations:** Department of Prosthodontics, School of Dentistry, Shiraz University of Medical Sciences, Shiraz, Fars, Iran; Medical University of South Carolina, UNITED STATES

## Abstract

**Objective:**

This study aimed to investigate the fracture resistance of one-piece screw-retained hybrid monolithic zirconia ceramic restorations in different implant angulations.

**Materials and methods:**

Three implant fixtures were embedded in acrylic resin blocks with 0°, 15°, and 25° angulations. For each group of implant angulations, 11 screw-retained one-piece monolithic zirconia restorations were made and bonded to the titanium inserts with a dual-cure self-adhesive resin. The complexes were screwed to the implant fixtures with titanium screws. The samples were thermocycled, subjected to compressive load, and fracture modes were recorded. One-way ANOVA and post hoc Tukey’s test were used for statistical analyses (α = 0.05).

**Results:**

One-way ANOVA showed the fracture resistance to be significanltly different among the study groups (*P* = 0.036). The 15° group was significantly more resistant than 0° (*P* = 0.031). However, the 25° group was not significantly different from the 15° (*P* = 0.203) and 0° groups (*P* = 0.624). Fractures occurred only on the restorations, not at the screw levels.

**Conclusions:**

Tilting the implant up to 15° improves the fracture resistance of one-piece screw-retained hybrid monolithic zirconia restorations; however, increasing the tilt to 25° would not yield restorations with significantly better fracture strength than the straight implants. Accordingly, when angulated implants are indicated in the esthetic zones, one-piece screw-retained hybrid monolithic zirconia ceramic restorations can be used with acceptable fracture strength.

## 1. Introduction

Tooth replacement with implants has become part of clinical practice because of its predictable outcomes [1–3]. A suitable treatment option to replace missing teeth is an implant-supported single crown with a 5-year survival rate of more than 90%. Restorations can be connected to the implant by either screws or cement [4]. In screw-retained restorations, the abutment may be separate from the restoration (two-piece) or combined as part of the fabrication procedure (one-piece) [4, 5]. Although cement-retained implant-supported restorations are more frequently used than screw-retained implant restorations, they have the major drawback of residual excess cement, which causes peri-implant diseases such as peri-implant mucositis and peri-implantitis [6].

Titanium abutments are considered the gold standard for durable implant-supported restorations [7–9]. However, the blue-grayish titanium abutments cause gingival discoloration in patients with a thin gingival biotype [10, 11]. This gray appearance can be masked through the intra-sulcular placement of prosthetic margins, which is likely to cause complications such as cement residues [12, 13].

Ceramic materials are preferred to metals since they cover the esthetic problems of porcelain-fused-to-metal restorations and titanium abutments; thus, fulfilling the esthetic demands of the patients [14, 15]. Zirconia ceramic is a dental material with excellent clinical features such as good mechanical properties, resemblance of natural appearance, minimal water solubility, no cytotoxicity, less bacterial adhesion, radio-opacity, and high corrosion resistance [15, 16]. Despite the enhanced material properties of yttria-stabilized zirconia ceramic (Y-TZP), failures of zirconia abutments have been observed clinically [17].

To address the esthetic concerns, zirconia frames are layered with esthetic ceramic veneer, whose chipping is the most frequent flaw of zirconia-ceramic restorations [8]. Therefore, clinicians recently advocated the use of zirconia ceramic as a monolithic material, which is shaped in the final anatomic and esthetic morphology of the tooth [8]. Additionally, an in-vitro study reported that cementing zirconia crowns on titanium abutments yielded less fracture-resistant restorations than zirconia crowns on zirconia abutments [18].

Concerning implant inclination, straight implants are usually preferred to facilitate simple and aesthetic rehabilitation [19]. Nonetheless, mostly due to the lack of bone in the height and width and sometimes because of incorrect surgical angulation in implant insertion, clinicians confront non-axial implants, which are difficult to restore properly based on functional needs and esthetics [19]. Evidence from a biomechanical investigation employing photo-elastic stress analysis indicates that axial placement of implants results in better stress distribution [19]. Implant fixtures are mostly installed with an angle >12° to the horizontal plane [20]. In such cases, the prosthetic abutment often needs to be tilted to facilitate a better emergence profile and enable prosthetic rehabilitation [21]. Compared with straight abutment positions, wider abutment angulations place more stress on the prostheses and surrounding bone [22]. Angulated abutments are available at 15 to 30 degrees of angulations [20]. The wall thickness on the side of an angulated abutment is directly related to the angulation degree [20]. Therefore, higher shear force and lower wall strength contribute to the risk of fracture [20].

To manufacture a monolithic restoration that can be finally screwed to the implant, zirconia abutment and zirconia restoration can be combined as a one-piece monolithic zirconia construction [4]. Hybrid abutment offers both strength and esthetics. The two components of a hybrid abutment are a titanium insert which is screwed to the implant body and contacts the implant platform and abutment screw, and a superstructure made of materials like lithium disilicate, zirconia ceramic, or resin-based composites [23]. The two components are extraorally assembled by either friction fit or bonding with resin-based cement. The superstructure is made through computer-aided design-computer-aided manufacturing (CAD-CAM) or heat-pressing techniques [23].

A systematic review showed favorable outcome of zirconium abutments in the incisive region, even in the canine-premolar region, in instances of unitary implant-supported rehabilitations [24]. Another systematic review concluded that 1-piece zirconia implants can be considered more fracture resistant than 2-piece zirconia implants. It was also found that abutment preparation of 1-piece zirconia implants should be avoided due to its negative impact on fracture resistance [25]. A study reported that tilting the implant apex to the lingual side significantly reduced the fracture strength of angle-corrected zirconia abutments [26]. Another study documented that the load-bearing performance of straight zirconia abutment was significantly better than angulated abutment only in absence of artificial aging [27]. On the contrary, a different study found that non-angulated monolithic zirconia abutments presented the lowest fracture resistance than those with 15° or 25° angulations [28].

The inclination of implants particularly in the esthetic zone can restrict the restoration of esthetical, functional, and mechanical necessities [27]. Due to the few studies about the fracture strength of one-piece screw-retained hybrid monolithic zirconia ceramic restorations and the controversial results, the purpose of this in vitro study was to further investigate the relation between implant angulation and the fracture resistance of one-piece screw-retained hybrid monolithic zirconia restorations under artificial aging to simulate the structural changes occurring in vivo and their fracture mode. The null hypothesis was that the fixture angulation would not affect the fracture strength of these restorations.

## 2. Materials and methods

### 2.1. Fabrication of one-piece screw-retained monolithic-zirconia restorations

**Fig 1:**
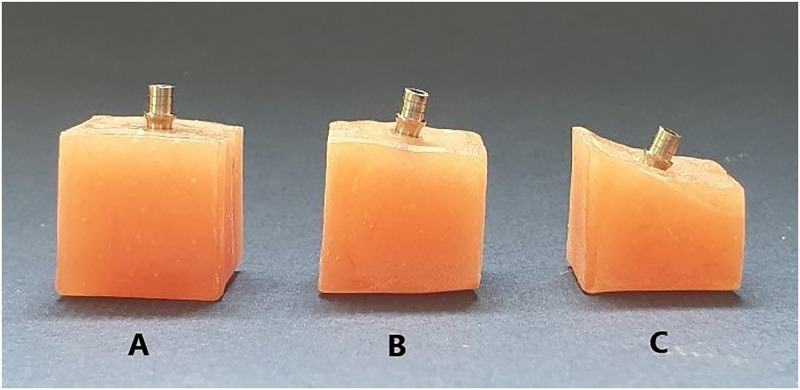
Different implant angulations: A) 0°, B) 15°, and C) 25°.

The study was designed to simulate implant rehabilitation of a maxillary first premolar. Auto-polymerizing acrylic resin blocks (Acropars Re; Marlic, Tehran, Iran) were prepared and implant fixtures (IF4010C ZrGEN; Anyone, Megagen, South Korea) were inserted in them up to the height of implant platforms to simulate the bone level placement of implants. Three implants (10×4 mm [length×diameter]) were used for 3 different angulations. A dental surveyor (Marathon-103; Saeyang, Daegu, Korea) was used to assure the exact inclination of the implant fixture to the long axis, which represented the occlusal force direction. The samples were divided into 3 groups based on the implant angulation with the original root configuration: 0° (following the original root configuration), 15° to the facial with respect to the implant crown (moderately deviated), and 25° to the facial with respect to the implant crown (more severely deviated) (Fig 1). In the angulated implants, the higher part of the implant platform was at the level of the upper part of the acrylic resin block.

**Fig 2:**
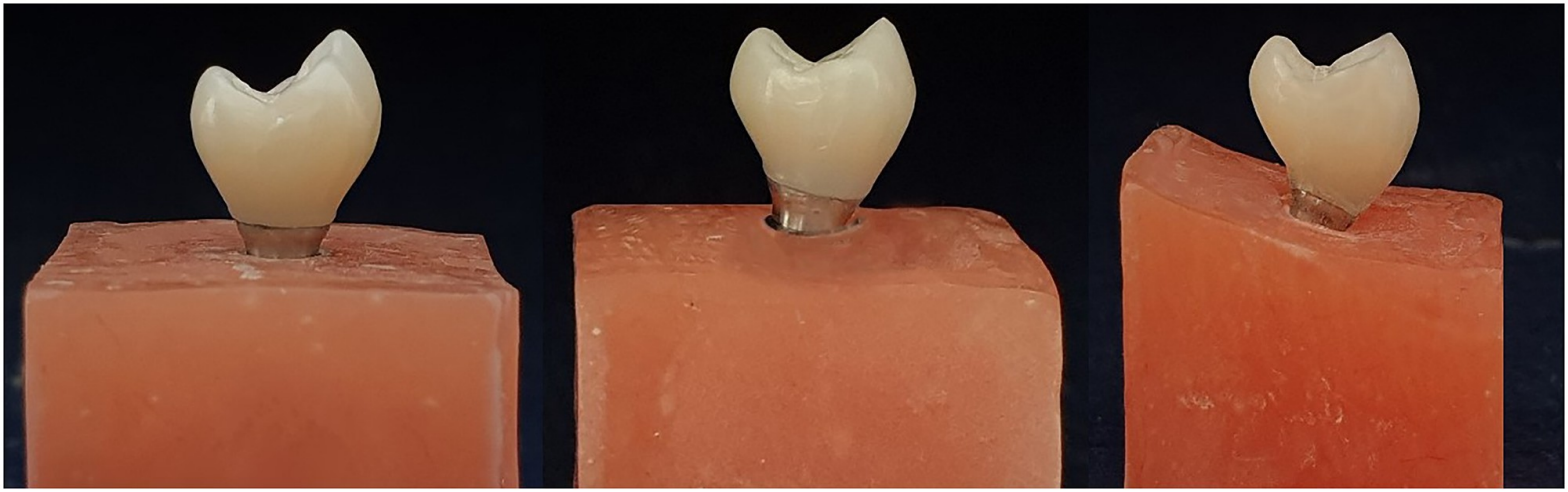
Three maxillary first premolar screw-retained one-piece monolithic zirconia restorations in 3 implant angulations.

Each of the three implant samples was scanned (Ceramill Map 400; Amann Girrbach GmbH) using Scan Abutments (AAOISR4009T Scan Abutment, Megagen, South Korea). Based on the 3 main implant angulations, 3 maxillary first premolar screw-retained one-piece monolithic zirconia restorations and their screw access hole were virtually designed in the CAD software program (Ceramill Mind CAD; version 3.5.6.1408, Amann Girrbach GmbH). A typodont maxillary first premolar (Columbia Dentiform Corporation, Long Island City, NY) was scanned to facilitate CAD for the zirconia restorations. Crown dimensions were kept as a constant factor simulating clinical conditions. Eleven samples per group were fabricated by milling partially-sintered monolithic zirconia blanks (ZIRAE; SHT Preshaded Zirconia Block, Nanjing Zirae Advanced Material Co, Nanjing, China) with a lab milling machine (Cori Tec 340i; imes-icor GmbH, Eiterfeld, Germany) (Fig 2).

The sample size was calculated according to a previous study [28], and 11 samples in each group were considered based on the power of 90%, type 1 error = 5%, the minimum difference of the 3 groups’ means = 184 (m1 = 718, m2 = 534; s1 = 93, and s2 = 133), calculated using the bellow sample size formula [29]:

$$n=\frac{(S_{1}^{2}+S_{2}^{2}){\left(Z_{{1-}_{2}^{\alpha }}-Z_{1-\beta }\right)}^{2}}{{\left(m1-m2\right)}^{2}}$$

To achieve fully-sintered zirconia restorations, all samples were sintered according to the manufacturer’s instructions in a high-temperature sintering furnace (Programat S1 1600; Ivoclar Vivadent, AG, Germany). Then, the fully-sintered one-piece monolithic zirconia restorations were bonded to the titanium implant inserts (AAOIPR4525 ZrGEN; Anyone, Megagen, South Korea). Therefore, the bonding surfaces of the titanium implant inserts were air-borne abraded with 50-μm aluminum oxide at a 10-mm distance for 10 seconds (0.4 MPa) [26]. The one-piece complexes were not air-abraded due to controversies about its plausible effect on the flexural strength and phase transformation of Y-TZP [30].

The screw holes of titanium inserts were sealed with heavy body impression material (Hydrorise Maxi Heavy; Zhermack, Germany) so that the excessive cement could not blockade access to the screw head. Then, the one-piece construction and titanium inserts were assembled and bonded by using a dual-cure self-adhesive resin cement (RelyX Unicem 2; 3M; Automix Self-Adhesive Resin) following the manufacturer’s instructions. The whole complex of one-piece restorations and titanium inserts were screwed to all implant fixtures with titanium screws. A manual torque wrench was used to torque the screws of one-piece monolithic zirconia restorations to the implant bodies and tightened at the manufacturer’s recommended torque force of 35 N-cm torque. The constructions were re-torqued after 10 minutes to prevent screw loosening [31].

**Fig 3:**
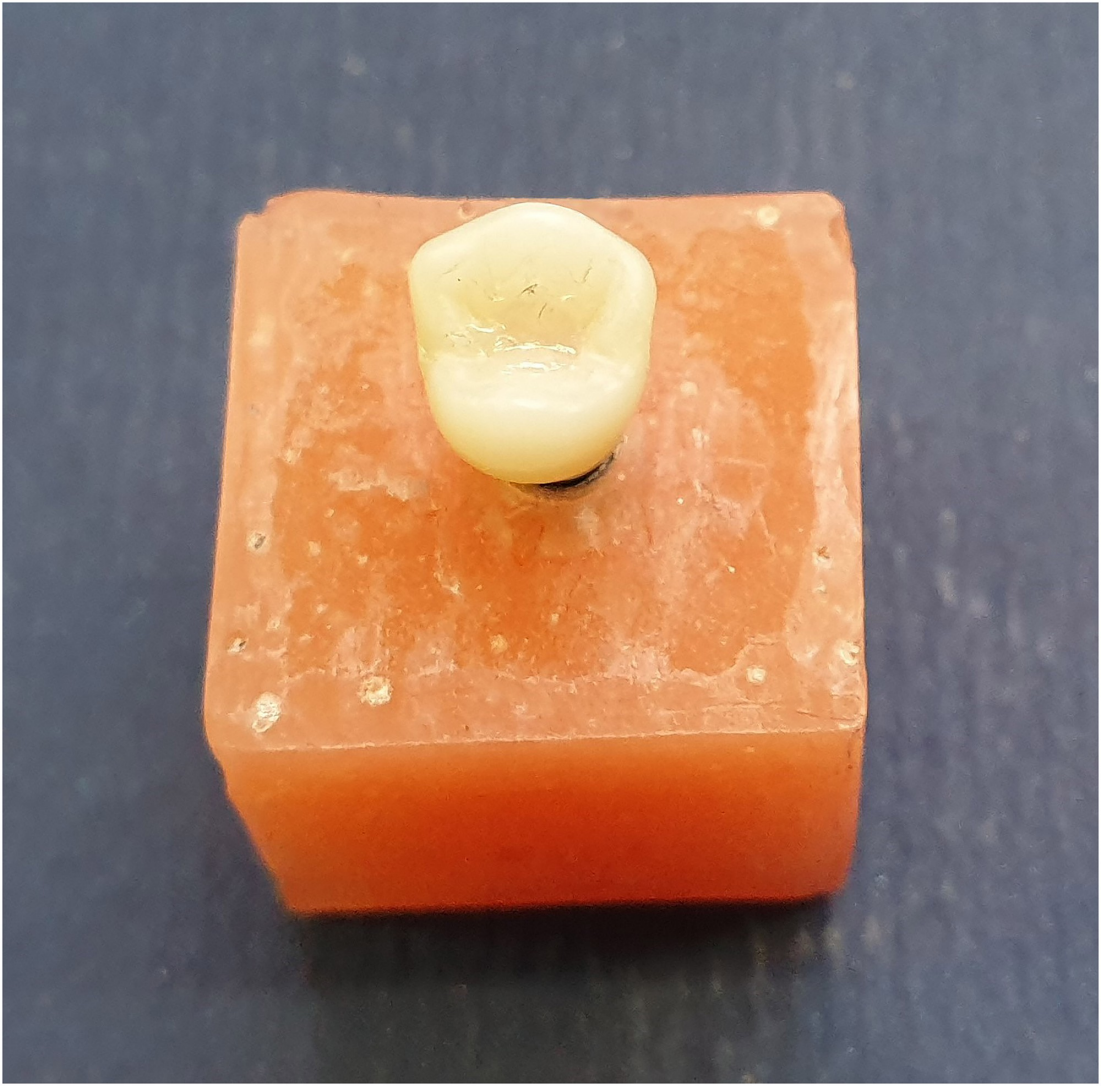
Final samples prepared for loading test.

All the access holes were filled with polytetrafluoroethylene film (Teflon tape) up to 2 mm apical to the occlusal plane as measured with a periodontal probe. After that, a bulk of composite resin (Esthet-X HD; Dentsply Sirona, USA) was placed, shaped, and light-cured for 40 seconds on the orifice to fill the screw access hole to the level of the occlusal plan (Fig 3) [32]. The procedures were all performed by a single skilled dental technician.

### 2.2. Thermocycling

To mimic the oral condition, the samples underwent 5000 thermal cycles between 5 and 50°C, with a dwell time of 30 seconds and a transfer time of 10 seconds. These 5000 cycles of cyclic thermal stressing corresponded to half a year of intraoral conditions [33]. All samples were visually inspected for incipient fracture and screw loosening. Signs of cracks or fractures of the ceramic as well as screw loosening were considered failures.

### 2.3. Evaluation of the fracture force

**Fig 4:**
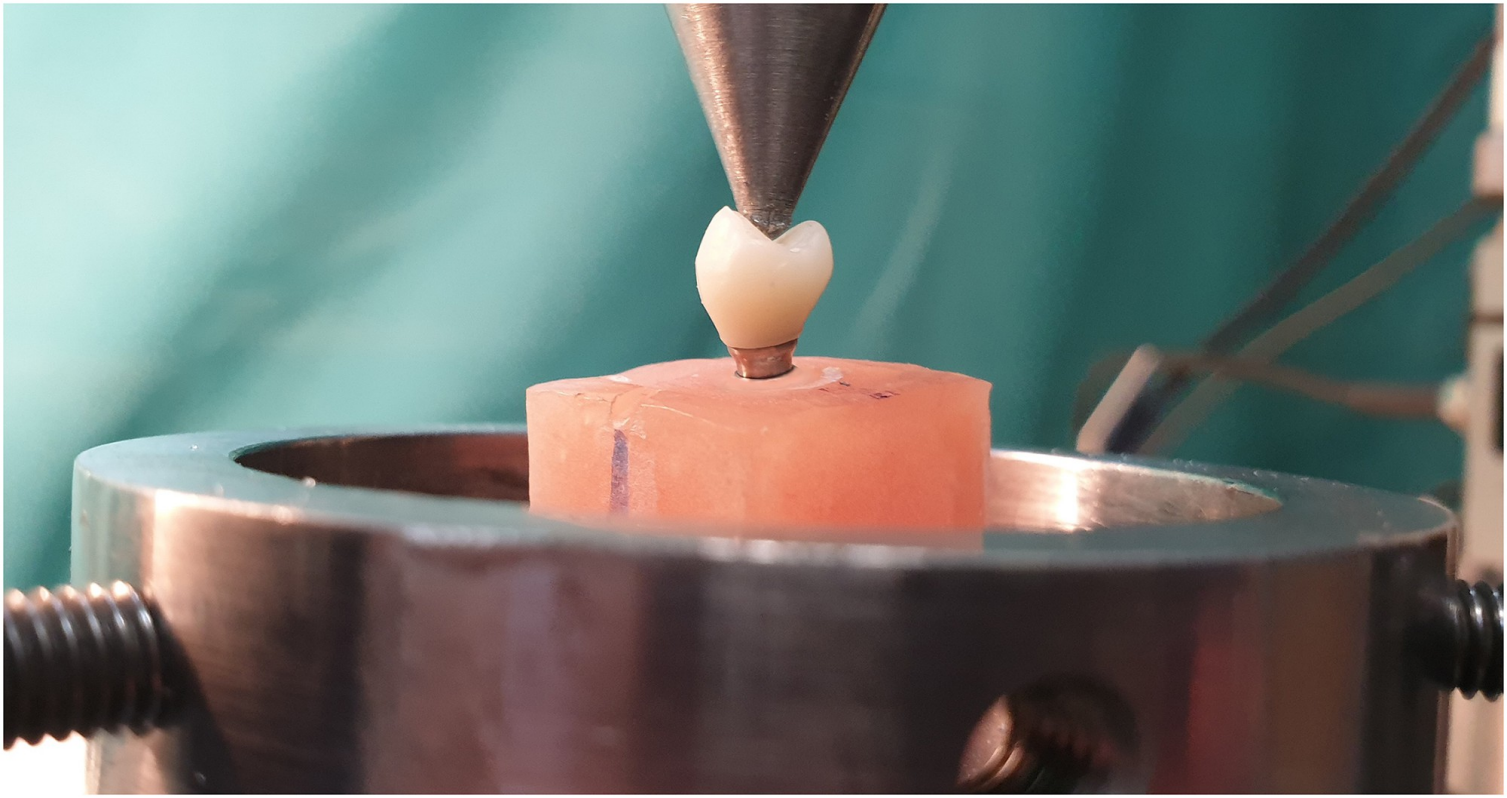
Compressive loading at a crosshead speed of 0.5 mm/min in a universal testing machine.

For mechanical testing, all samples were oriented to create a perpendicular relationship between the long axis of the simulated maxillary first premolar crown and the occlusal plane. Attempts were made to meticulously direct the compressive force centrally utilizing a force applicator 3 mm in diameter placed along the central fossae of restorations. Perpendicular compressive force to the central fossa of crowns was chosen for the fracture test as it simulated the most common force clinically applied to these teeth [34]. A compressive load was applied at a crosshead speed of 0.5 mm/min by a Universal Testing Machine (Shimadzu Autograph AG-50kNE, Shimadzu Co., Ltd, Japan) [32] (Fig 4).

The loading went on until fracture was visibly detected with a sudden drop in the force as seen in the graph. The force required (N) to cause fracture of each sample was recorded. The samples were optically monitored and apparent failures were documented. The fracture mode was classified based on scanning electron micrographs (SEM; ×14–1000 magnifications; working distance: 9–13 mm; voltage: 20 kV; low-vacuum; TESCAN VEGA3, Hong Kong) at ×30, ×500, and ×1000 magnifications.

### 2.4. Statistical analysis

Statistical analyses of data were done through the SPSS software program (SPSS Inc; version 20, Chicago, Illinois, USA). The mean values and standard deviations were calculated in each group. Shapiro-Wilk and Levene’s tests were used to check the normality and equalization of variances. One-way ANOVA and Tukey’s post hoc pairwise multiple comparisons were used to analyze and determine statistically significant differences among the angulation groups (α = 0.05).

## 3. Results

**Fig 5:**
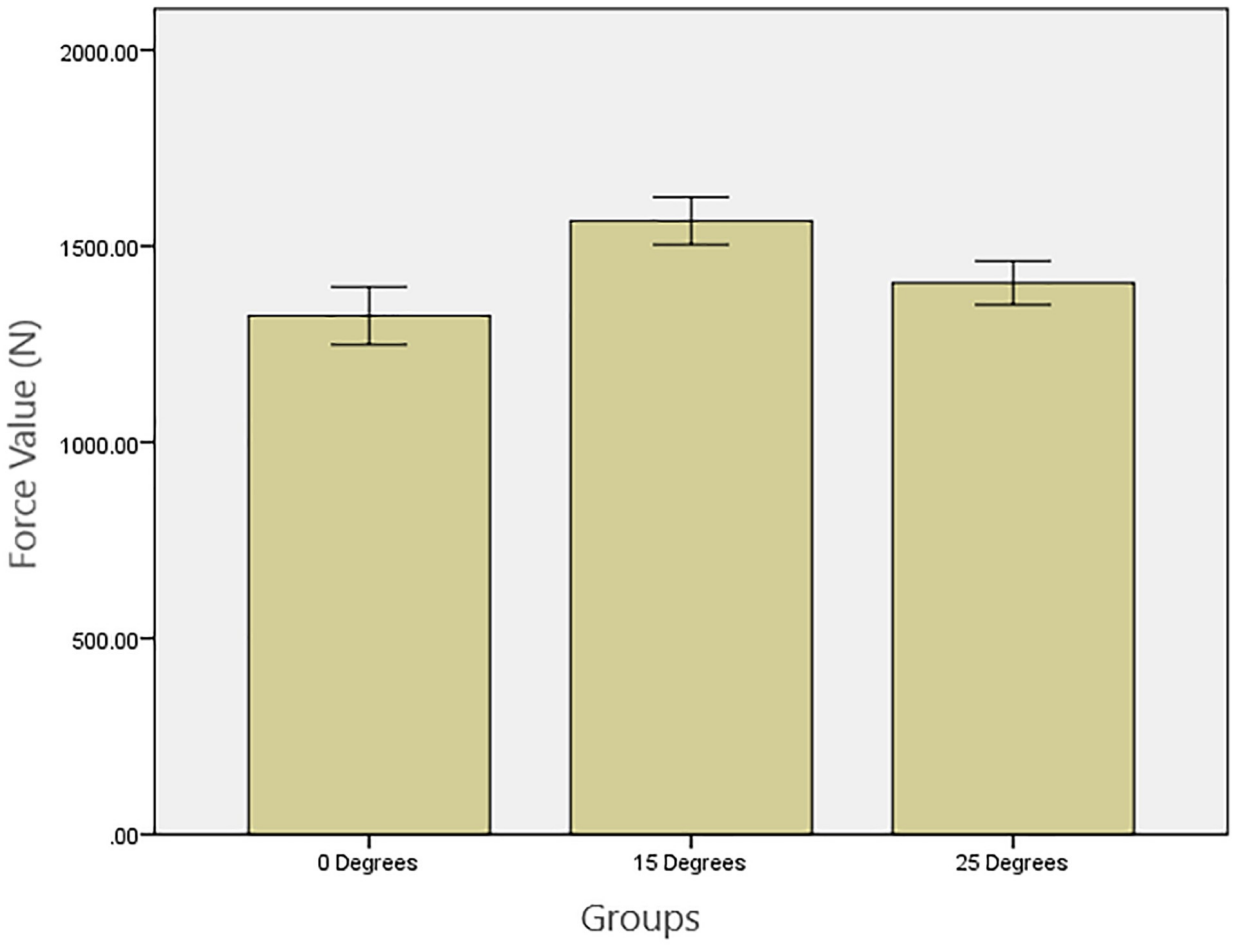
Mean fracture strength and standard deviation (N) for different implant angulations.

As displayed in Table 1, the P value for Shapiro-Wilk and Levene’s tests approved the normal distribution and homogeneity of variances among the 3 groups. The results of one-way ANOVA showed significantly different fracture resistances among the 3 angulation groups (*P* = 0.036). The mean and standard deviation of fracture resistance are presented in Table 2 and Fig 5. Tukey’s post hoc test showed that the fracture resistance of 15°-angulated restorations was significantly higher than the straight restorations (*P* = 0.031). The fracture resistance of the restorations with 25° angulation was higher than the straight restorations, although the difference was not statistically significant (*P* = 0.624). Nor was the difference between the 15° and 25° groups statistically significant (*P* = 0.203).

**Table 1:** P values for Shapiro-Wilk and Levene’s tests.

Groups	Shapiro-Wilk	Levene’s tests
0°	0.264	0.913
15°	0.224	
25°	0.520	

**Table 2:** Means ± standard deviations of load at fracture (N) and multiple comparisons among the groups.

	0° Group (n = 11)	15° Group (n = 11)	25° Group (n = 11)	P value^a^
Load at fracture (N)	1322.10 ± 244.65	1564 ± 200.34	1406.95 ± 185.56	0.036

^a^ANOVA test. P value = 0.031, 0.624, and 0.203 for comparing the 0° and 15° groups, 0° and 25° groups, and 15° and 25° groups, respectively.

**Fig 6:**
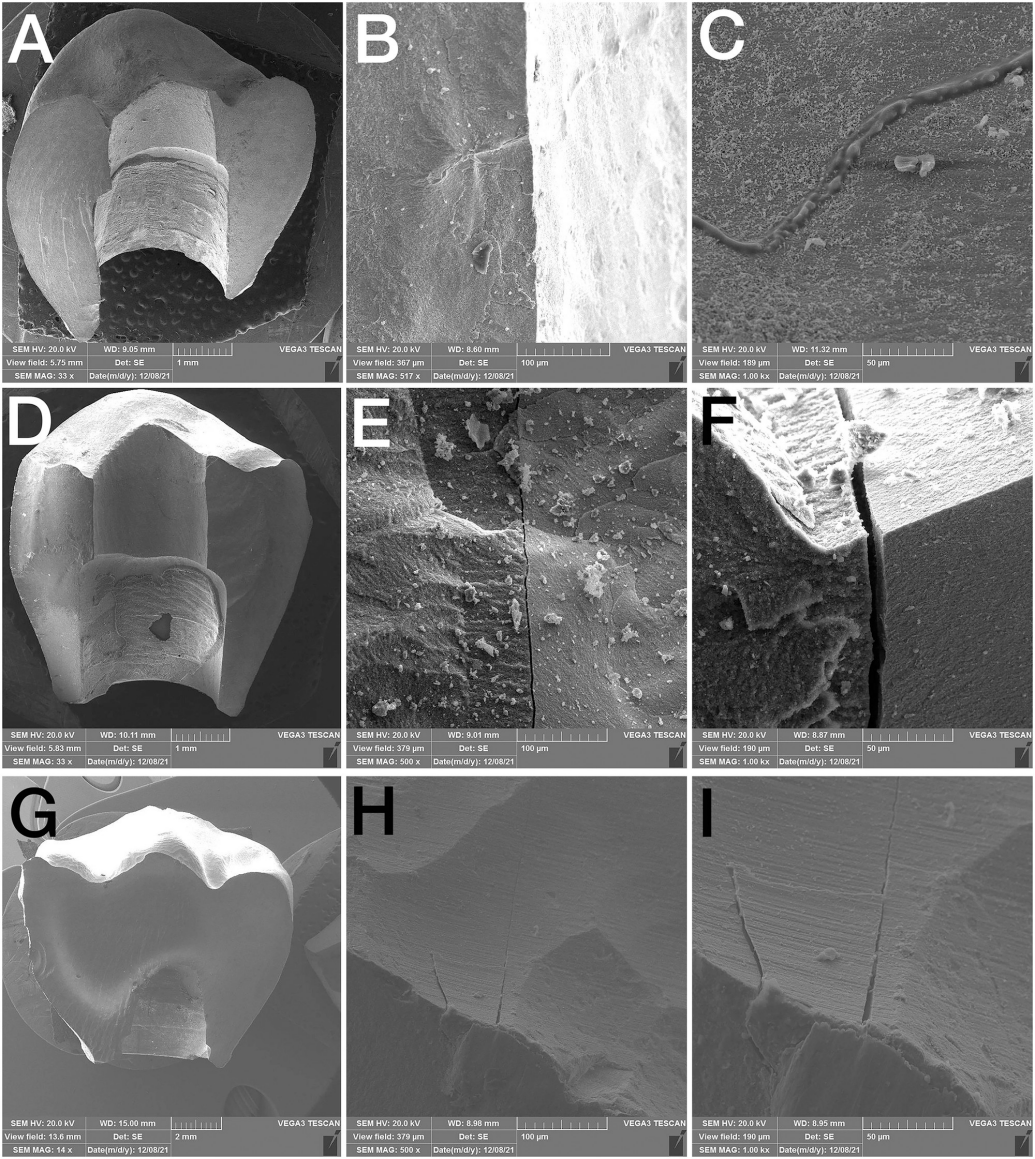
SEM overview of fractures at different magnifications; A, B, C: 0° group, D, E, F: 15° group; G, H, I: 25° group.

The titanium inserts of all restorations remained attached to their corresponding implant fixtures after testing the load-to-fracture and all monolithic restorations were split into two pieces. The SEM analysis showed that the fractures began at the occlusal surface of one-piece monolithic zirconia restorations across their screw channel hole for all the samples (Fig 6).

## 4. Discussion

The null hypothesis was rejected since implant angulation significantly affected fracture resistance. Restorations with a 15° long axis deviation from the implant axis had the highest fracture resistance among all other angulation groups and were significantly more fracture resistant than 0°. But the fracture resistance was not significantly different between the 15° and 25° groups and 0° and 25° deviations groups.

Clinically, to correct the non-axial implants, abutments are more often angulated between 5° and 30°. Angulated abutments are reported to have a high survival rate [35]. In the present study, 0° angulation demonstrated the ideal inclination of restoration, and 15° and 25° were the usual inclinations to correct the non-axial angulations of fixtures. In addition to loading angle, loading point, aging treatments, and implant angulation were incorporated into the design to simulate common intraoral conditions and limitations caused by the availability of bone and surgical errors in the insertion of implants.

**Fig 7:**
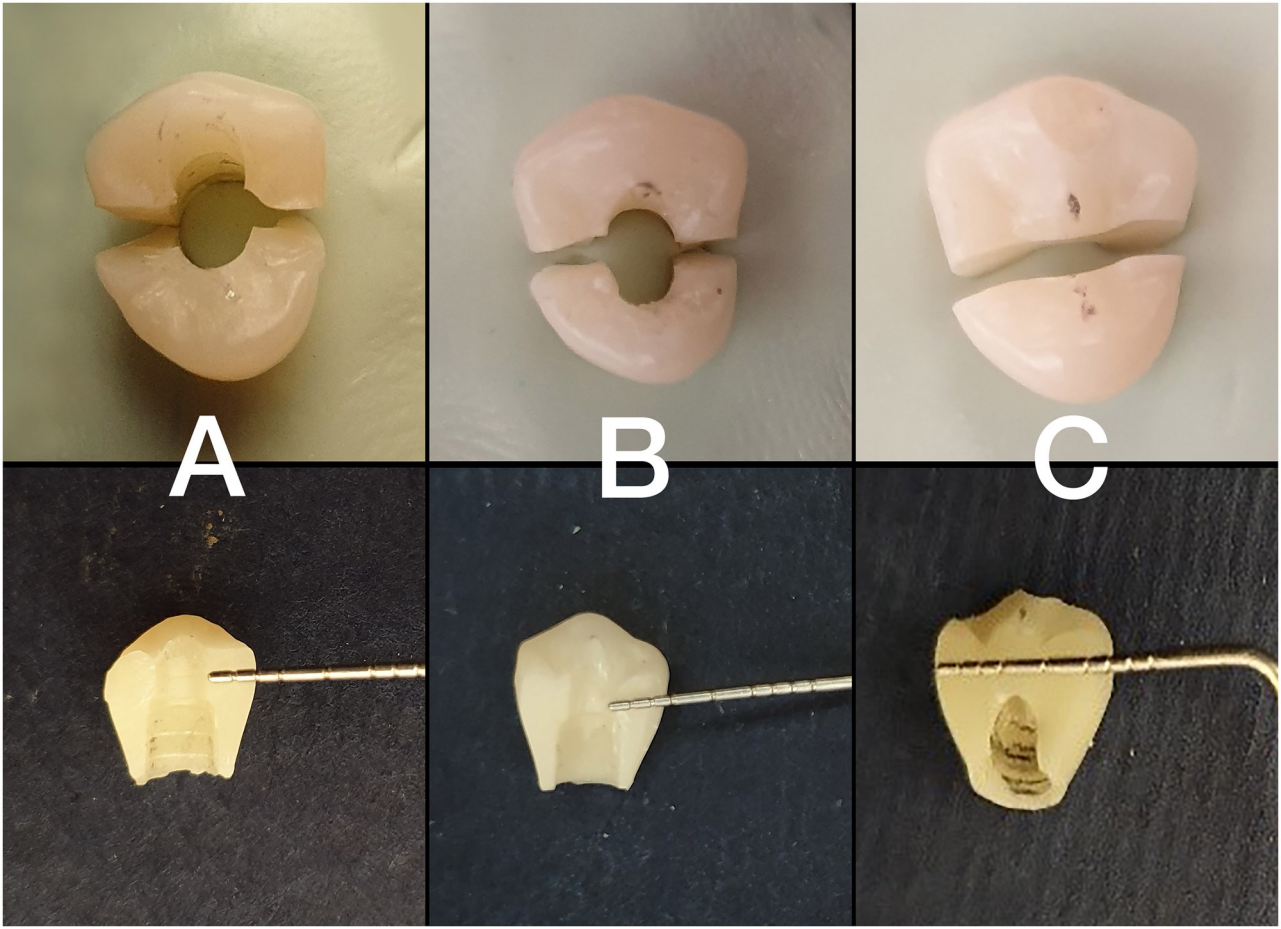
Peripheral zirconia thickness based on fracture location (A: 0° group, B: 15° group, C: 25° group).

Like most brittle materials, zirconia ceramic has a high compressive and low tensile strength [36], appropriate ceramic thickness may decrease internal stress and reduce mechanical failure [36]. The current study showed that the restorations with 0° inclinations had the lowest fracture strength because the point of force entry was exactly above the screw access hole that had the lowest zirconia peripheral thickness (2 mm). With 15° angulation, the force entry point was slightly far from the screw access hole, consequently, increased maximum restoration thickness in that area (3 mm) compensated for the effect of the angular force in reducing fracture strength. As the result showed, fracture resistance of restorations with 25° angulations was even more than 0° but less than 15° (not statistically significant but clinically important), indicating that the thickness (8 mm) had a more prominent effect on the fracture strength than the angular force (Fig 7).

Katsavochristou et al. [28] investigated the fracture resistance and performance of zirconia implant abutments with different angulations. They concluded that the implant-to-abutment angulation of 15° yielded significantly higher fracture resistance than 0° and 25°; which was in line with the present study. It was mentioned that based on standard deviations, the data for group 0° were much more reliable. In general, the standard deviation may rely on the intrinsic characteristics of the material, manufacturing factors during abutment milling, and the operator’s error when using the universal testing machine. Moreover, the samples with 25° angulation were more fracture-resistant than the straight ones (0°); which is in agreement with the current findings. However, unlike the present study, this difference was statistically significant in Katsavochristou’s study probably due to the different study designs, size, shape, and location of the restoration [37].

In line with the current study, Nothdurft et al. [27] found that in the absence of artificial aging, the restorations with angulated (20°) zirconia custom abutments were more fracture-resistant than those with straight abutments. Probably the bending forces are lower in the area of the apical hexagon, co-localized with the thinnest portion of the angled abutment. Saker et al. [20] assessed the impact of implant platform diameters on the ultimate force of failure of zirconia abutments with different angulations in the anterior zone. They observed that fracture resistance was similar for the straight and angulated zirconia abutments on 3-mm and 3.5-mm platforms; yet, significantly less for angulated zirconia abutments on the 4.5-mm platform. It is noteworthy that in Saker’s study, there was a 30° angle between the crown’s long axis and load direction, which can justify their different results.

On the contrary, in Albosefi et al.’s study [38], a compromised implant position requiring 15° angulated zirconia custom abutments had a lower fracture load than the straight ones. Such a difference can be attributed to the less bulk thickness in zirconia abutments compared with the custom one-piece zirconia restorations which were used in the current study.

Up to now, almost all similar studies concluded that wider angulation decreases the fracture resistance of restorations [26–28, 39, 40]. Nonetheless, no recent study has assessed zirconia restorations in combination with the abutment as a one-piece construction, like what actually happens in the mouth.

### 4.1. Angulation and fracture mode

The fracture resistance of implant abutments is highly related to the material of samples, whether they are zirconium dioxide abutments with a titanium alloy carry base [37] or real all-ceramic components. Regarding the currently evaluated one-piece zirconia construction with a titanium alloy carry base, the SEM analysis showed that the fracture pattern began from the occlusal surface and radiated across the bulk of the monolithic zirconia restoration along the screw access hole to the titanium alloy carry base, starting with a cohesive fracture far above the titanium inserts in all samples, splitting them into two pieces (Fig 5). This observation revealed that the various buccal dimensions caused by different screw hole locations could not affect the fracture mode, but only the fracture resistance.

Ellakwa et al. [39] detected specific large fractographic patterns such as arrest lines, Wallner’s lines, and large twist and wake hackle lines, by using SEM. Having examined these characteristics, they figured out that crack propagation originated from the occlusal surface of the crowns and radiated toward the gingival margin. This might be attributed to the different stresses generated according to the abutment angulation and the direction of occlusal compressive load. Their results are similar to that of the present study probably because of similar study design such as propriety thickness and the use of titanium inserts.

In the meantime, some studies reported that all zirconia abutments (with zirconia connections) typically fractured at the cervical part of the abutment near the screw and platform of the implant [27]. It suggested the hex as the weakest part of the entire abutment and the site for crack initiation, because the inherently brittle zirconia is sensitive to tensile forces and breakage, especially in thin dimensions [26]. Probably the use of a titanium alloy carry base instead of all zirconia connections in the current study influenced the fracture mode in monolithic zirconia restorations.

Among the limitations was the in-vitro nature of the study. Moreover, this study did not evaluate the effect of mechanical cycling on fracture resistance. Studies are suggested to compare the fracture resistance of one-piece and two-piece zirconia restorations in different angulations.

## 5. Conclusions

Within the limitations of this study, the following conclusions can be drawn:

1. One-piece screw-retained hybrid monolithic zirconia restorations with 15° implant-restoration angulation are significantly more fracture-resistant than 0°.
2. Increasing implant-to-restoration angulation from 0° up to 25° insignificantly improves the fracture-resistances of one-piece hybrid monolithic zirconia restorations.
3. All samples were cohesively fractured starting from the occlusal surface near the force entry point.
4. When the use of angulated implants is indicated in the esthetic zones, one-piece screw-retained hybrid monolithic zirconia restorations would not negatively affect the fracture strength of restoration, it rather increases it.

## Supporting information

S1 Table Raw data of all the study groups. (XLSX)
